# D936Y and Other Mutations in the Fusion Core of the SARS-CoV-2 Spike Protein Heptad Repeat 1: Frequency, Geographical Distribution, and Structural Effect

**DOI:** 10.3390/molecules26092622

**Published:** 2021-04-30

**Authors:** Romina Oliva, Abdul Rajjak Shaikh, Andrea Petta, Anna Vangone, Luigi Cavallo

**Affiliations:** 1 Department of Sciences and Technologies, University Parthenope of Naples, Centro Direzionale Isola C4, I-80143 Naples, Italy; 2 Kaust Catalysis Center, Physical Sciences and Engineering Division, King Abdullah University of Science and Technology (KAUST), Thuwal 23955-6900, Saudi Arabia; abdulrajjak.shaikh@kaust.edu.sa (A.R.S.); luigi.cavallo@kaust.edu.sa (L.C.); 3 Dipartimento di Informatica ed Applicazioni, University of Salerno, Via Papa Paolo Giovanni II, I-84048 Fisciano, Italy; anpetta@unisa.it; 4 Roche Innovation Center Munich, Pharma Research and Early Development, Large Molecule Research, Nonnenwald 2, 82377 Penzberg, Germany; anna.vangone@roche.com

**Keywords:** COVID-19, spike protein, mutations, molecular dynamics, infectivity

## Abstract

The crown of the severe acute respiratory syndrome coronavirus 2 (SARS-CoV-2) is constituted by its spike (S) glycoprotein. S protein mediates the SARS-CoV-2 entry into the host cells. The “fusion core” of the heptad repeat 1 (HR1) on S plays a crucial role in the virus infectivity, as it is part of a key membrane fusion architecture. While SARS-CoV-2 was becoming a global threat, scientists have been accumulating data on the virus at an impressive pace, both in terms of genomic sequences and of three-dimensional structures. On 15 February 2021, from the SARS-CoV-2 genomic sequences in the GISAID resource, we collected 415,673 complete S protein sequences and identified all the mutations occurring in the HR1 fusion core. This is a 21-residue segment, which, in the post-fusion conformation of the protein, gives many strong interactions with the heptad repeat 2, bringing viral and cellular membranes in proximity for fusion. We investigated the frequency and structural effect of novel mutations accumulated over time in such a crucial region for the virus infectivity. Three mutations were quite frequent, occurring in over 0.1% of the total sequences. These were S929T, D936Y, and S949F, all in the N-terminal half of the HR1 fusion core segment and particularly spread in Europe and USA. The most frequent of them, D936Y, was present in 17% of sequences from Finland and 12% of sequences from Sweden. In the post-fusion conformation of the unmutated S protein, D936 is involved in an inter-monomer salt bridge with R1185. We investigated the effect of the D936Y mutation on the pre-fusion and post-fusion state of the protein by using molecular dynamics, showing how it especially affects the latter one.

## 1. Introduction

Coronavirus Disease 2019 (COVID-19) is caused by the severe acute respiratory syndrome coronavirus 2 (SARS-CoV-2), which is also referred to as human coronavirus 2019 (hCoV-2). SARS-CoV-2 is a novel virus belonging to the β genus coronaviruses, which also include two highly pathogenic human viruses identified in the last two decades, known as the severe acute respiratory syndrome coronavirus (SARS-CoV) and the Middle East respiratory syndrome coronavirus (MERS-CoV) [1,2,3].

Coronaviruses are named after the protruding spike (S) glycoproteins on their envelope, giving a crown shape to the virions [4]. Of the four structural proteins of coronaviruses: S, envelope (E), membrane (M), and nucleocapsid (N), the S protein is the one playing a key role in mediating the viral entry into the host cells [5,6,7], making it one of the main targets for the development of therapeutic drugs and vaccines [8,9,10,11,12,13,14]. Comprised of two functional subunits, S1 and S2, it first binds to a host receptor through the receptor-binding domain (RBD) in the S1 subunit and then fuses the viral and host membranes through the S2 subunit [7,15]. In the pre-fusion conformation, the SARS-CoV-2 S protein forms homotrimers protruding from the viral surface, where its RBD binds to the angiotensin-converting enzyme 2 ACE2 receptor on the host cell surface [1] (like the SARS-CoV homolog [16], and differently from MERS-CoV S, which recognizes a different receptor, the dipeptidyl peptidase 4 [17]. Receptor binding and proteolytic processing by cellular proteases then cause S1 to dissociate and S2 to undergo large-scale conformational changes toward a stable structure, bringing viral and cellular membranes into close proximity for fusion and infection [7,15,18].

While the outbreak of COVID-19 was rapidly spreading all over the world, affecting millions of people and becoming a global threat, laboratories worldwide promptly started to sequence a large number of SARS-CoV-2 genomes. All the available genomic data is accessible through the Global Initiative on Sharing All Influenza Data (GISAID) website, which is an invaluable open access resource [19,20]. Simultaneously, crucial structural knowledge has been achieved on SARS-CoV-2, especially regarding the S protein. On 15 March 2021, ≈280 experimental 3D structures of the SARS-CoV-19 S protein were available from the Protein Data Bank (PDB) [21]. These include the structure of the SARS-CoV-2 S protein in the pre-fusion conformation, also bound to the ACE2 receptor [22,23,24,25,26,27,28], and of the post-fusion core of its S2 subunit in the post-fusion conformation [29].

After the proteolytic processing, in the post-fusion conformation, the S protein HR1 and HR2 motifs interact with each other to form a six-helix bundle (6-HB), which promotes initiation of the viral and cellular membranes fusion [7,15,18]. The 6-HB formation is a conserved and critical mechanism for viral fusion and entry, shared by all coronaviruses. The HR1 “fusion core” is named after its role in giving many interactions with HR2 in the post-fusion conformation, thus, playing a key role in the virus infectivity, and being a target for inhibitors of the SARS-CoV-2 fusion capacity [29,30,31]. On these bases, we decided to investigate the frequency and possible structural effect of the mutations accumulated over time in this crucial, functional motif.

On 15 February 2021, genomic sequences of SARS-CoV-2 available from GISAID had overcome 550,000. A search in such a resource showed a variable occurrence of mutations at different positions of the HR1 fusion core, with mutant D936Y being the most frequent, with 1296 occurrences, particularly in some European countries, especially Finland and Sweden. Among the identified HR1 mutants, it is also the mutant expected to have the most significant structural consequences, as D936 is involved in an inter-monomer salt bridge in the post-fusion assembly. Therefore, we performed a comparative study of the wild-type S protein and the D936Y mutant, both in their pre-fusion and post-fusion conformations by molecular dynamics (MD). Results of the MD simulations helped us illuminate the effect of the mutation on the protein structure and dynamics.

We also investigated the structural basis, both in the pre-fusion and post-fusion conformation as well as sequencing dates and geographical distribution of the other two most frequent HR1 mutations, S939F and S929T, with 1108 and 467 occurrences, respectively. For the pre-fusion conformation, a structure with one RBD in the up and two RBDs in the down position was considered, which has been recently proposed, based on molecular dynamics analyses [32], to be significantly more stable than the structure with two RBD in the up position, and, therefore, likely to be the protein state observed prior to interacting with the cell surface [33].

Considering the impressive pace at which new SARS-CoV-2 sequences are obtained and collected, we have also set up a web application providing a periodic update of mutations in the S protein HR1 fusion core (at https://www.molnac.unisa.it/BioTools/cov2smt/index.php) (accessed on 18 April 2021).

## 2. Results

### 2.1. Identification of the HR1 “Fusion Core” Mutations

**Figure 1:**
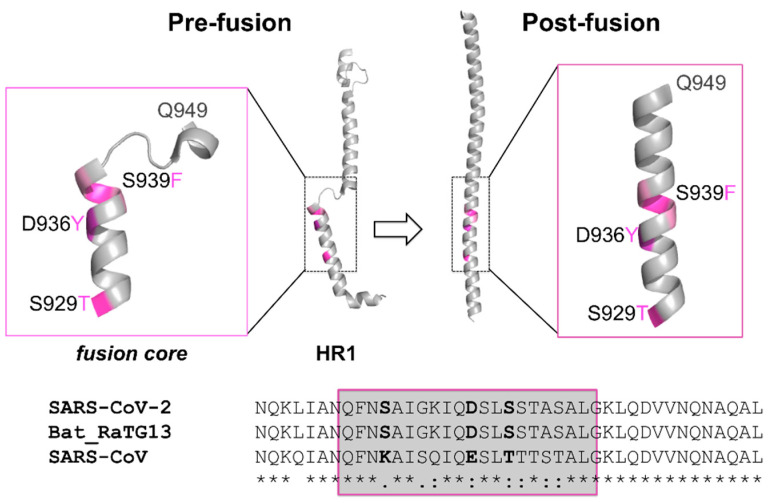
Structural and sequence location of the reported mutations. Top: Cartoon representation of SARS-CoV-2 S protein HR1 and its fusion core (insets) in the pre-fusion and post-fusion conformations (PDB IDs: 6VSB and 6LXT). Discussed mutations are colored in purple and labelled. Q949, at the end of the fusion core, is also labeled. Bottom: Sequence alignment of the HR1 fusion core (framed) and 10 residues up-stream and down-stream in the S protein of SARS-CoV-2, bat coronavirus RaTG13 (protein_ID: QHR63300.2), and SARS-CoV (protein_ID: AAP13441.1).

The HR1 of coronaviruses S proteins undergoes one of the most notable rearrangements within the protein between the pre-fusion and post-fusion conformations. In the post-fusion conformation, it experiences a refolding of the pre-fusion multiple helices and intervening regions into a single continuous helix (Figure 1). As already mentioned, three of these long helices then form a 6HB with three HR2 helical motifs [18,29,30]. The HR1 and its “fusion core” particularly play a crucial role in the virus infectivity.

On 15 February 2021, we downloaded all the SARS-CoV-2 genomic sequences from the GISAID resource, extracted from them 415,673 complete S protein sequences, and identified all the point mutations occurring in the S929-Q949 region (see Methods). The identified mutations with the relative number of occurrences are reported in Table S1. Only the most frequent mutations are reported in Table 1.

Most of the positions, such as 931, 933 to 935, 937, 944-945, and 948-949, were virtually unaffected by mutational events, with a maximum mutation rate of 0.005%. Positions 930, 937, 941, and 946-947 were also little affected, with a mutation rate below 0.02%. Positions 932, 938, 940, 942-943 had a mutation rate between 0.025% and 0.055%. Positions featuring the higher number of mutations were 929, 936, and 939, which are all located in the N-terminal half of the HR1 fusion core and featuring a mutation rate above 0.1%. Starting from position 929, S929 was found to mutate to threonine in 467 sequences and to asparagine/arginine/glycine in 60/5/1 sequences.

As for position 936, D936 was mutated to tyrosine in 1296 sequences and to asparagine/histidine/valine/glycine/glutamate/glutamine/alanine/serine in 148/125/44/24/17/3/1/1 sequences.

Finally, S939 was mutated to phenylalanine in 1108 sequences and to tyrosine/leucine/alanine in 5/3/1 sequences.

### 2.2. Geographical Distribution of the HR1 “Fusion Core” of Most Frequent Mutations

**Figure 2:**
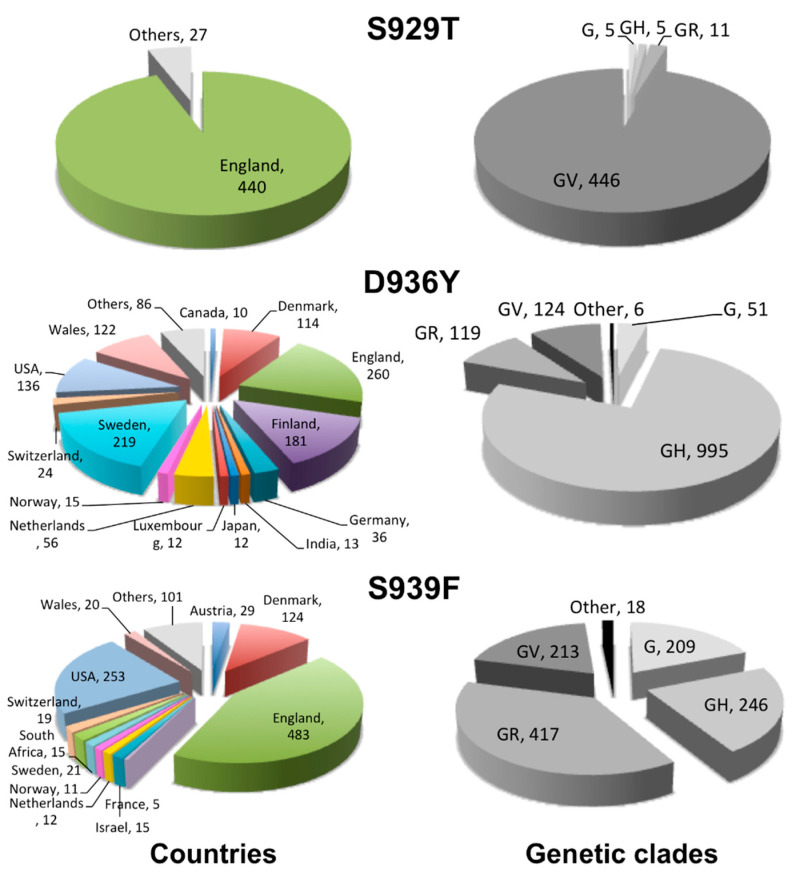
Countries and genetic clades. Pie chart visualization of the geographical distribution (left panel) and phylogenetic classification (right panel) of sequences presenting the S929T, D936Y, and S939F mutations.

The geographical distribution, per country, of the investigated mutations is reported in Figure 2. The first occurrence of the S929T mutation was deposited in GISAID on 18 April 2020, which is sequenced in Canada. On 15 February, however, the large majority of its occurrences was reported from England (440 over 467, corresponding to 94%). The remaining 27 occurrences were also mostly sequenced in Europe, with only 5 overall occurrences from USA and Canada, 2 from Australia, and 1 from South Africa.

The first occurrence of the D936Y mutation was, instead, deposited in GISAID on 8 March 2020, which was sequenced in Sweden. On 15 February 2021, occurrences have been reported from 48 countries. However, Sweden confirms itself as the country with the higher occurrences of such a mutation (in 219 sequences, representing 17% of the total). The other four European countries contributed, together with Sweden, 60% of all the occurrences. These countries are England, Finland, Wales, and Denmark, and reported 260 (20%), 181 (14%), 122 (9.4%), and 114 (8.8%) occurrences, respectively. USA also contributed a significant number of occurrences (136 occurrences or 10%). The remaining 30% of occurrences were mainly sequenced in European countries, the Netherlands (56), Germany (36), Switzerland (24), Norway (15), Luxembourg (12), Scotland (5), Austria (5), and others, as well as in India (13), Japan (12), Canada (10), Mexico (7), Singapore (5), etc. (for a complete list, see the web site: https://www.molnac.unisa.it/BioTools/cov2smt/index.php) (accessed on 28 April 2021).

Notably, the total number of occurrences of the D936Y mutation amounted to 17% of all the 1089 sequences available from Finland and to 12% of all the 1768 sequences available from Sweden.

The first occurrence of the S939F mutation was deposited in GISAID on 25 February 2020 from the United Arab Emirates. On 15 February 2021, it was spread in 44 countries, especially western ones. Three countries represented together 66% of all the occurrences. These countries are England, USA, and Denmark, having reported 483 (37%), 253 (20%), and 124 (9.6%) occurrences. Over 10 occurrences of the mutation were also reported from other European countries: Austria (29), Sweden (21), Wales (20), Switzerland (19), the Netherlands (12), and Norway (11), but also from Israel (15) and South Africa (15). Two more occurrences of the mutation have been reported from the United Arab Emirates between May and June 2020.

### 2.3. Clade Association of the HR1 “Fusion Core” of Most Frequent Mutations

The distribution of the mutations in high-level phylogenetic groupings, or genetic clades, is plotted in Figure 2. As a reminder, the G/GH/GR/GV clades are among the latest out of eight genetic clades reported in GISAID (S, L, V, G, GH, GR, GV, GRY) [34]. The G clade carries the D614G mutation, now globally dominant, accompanied by other mutations upstream the S protein gene (C241T, C3037T). In addition, the GH clade presents the NS3-Q57H mutation, the GR clade presents the N-G204R mutations, and GV clade presents the S-A222V mutation.

The three reported mutations HR1 are clearly associated with the late G/GH/GR/GV clades. In particular, S929T is mainly associated with the GV clade and D936Y is mainly associated with the GH clade, while S939F is roughly equally associated with the GR, GH, GV, and G clades.

### 2.4. Sequence Conservation among Similar Viruses

All the amino acids in the three positions more prone to mutation in the SARS-CoV-2 S protein HR1 fusion core are conserved in the bat coronavirus RaTG13 S protein (sharing an overall sequence identity of 97% with SARS-CoV-2 S protein), while all of them are mutated in the SARS-CoV S protein (overall, 76% sequence identical to the SARS-CoV-2 homolog) (see Figure 1). In particular, S929 is a lysine in SARS-CoV, while D936 is substituted by a glutamate and S939 by a threonine. It has been proposed that the SARS-CoV-2 HR1 mutations as compared to SARS-CoV may be associated with enhanced interactions with HR2, further stabilizing the 6-HB structure and maybe leading to increased infectivity of the virus [29]. In this context, it is noteworthy that the point mutations we are discussing did not restore the corresponding SARS-CoV amino acid.

### 2.5. Effect of the Mutations on the Protein Pre-Fusion Conformation

**Figure 3:**
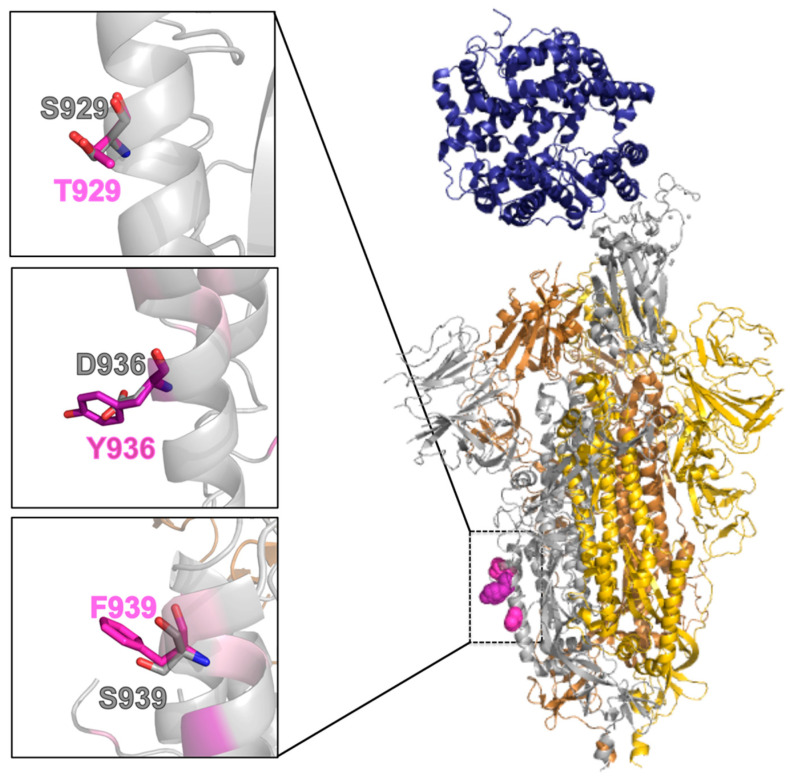
Mutants in the pre-fusion conformation. Right: Cartoon representation of the SARS-CoV-2 S protein in its pre-fusion trimeric conformation (the three monomers are colored in silver, gold, and copper, PDB ID: 6VSB), with the structure of the RBD bound to the ACE2 receptor (in blue, PDB ID: 6M0J) superimposed on its chain A. The most frequent mutations in the HR1 fusion core in GISAID on 15 February are colored purple and shown as a “dots” representation for chain A. Left: Focus on the structural context of each wild-type residue (silver sticks) and corresponding mutant (purple sticks).

In the pre-fusion conformation, the most mutated positions are located on the second of four non-coaxial helical segments composing the HR1 (Figure 1). They are all exposed to the solvent (Table 2), and can be modelled as larger residues without causing any structural strain (see Figure 3). These mutations are not expected to cause relevant changes in the pre-fusion structure. However, they could have a destabilizing effect as a consequence of posing large aromatic residues, at positions 936 and 939, in direct contact with the solvent instead of a charged aspartate or polar serine residue.

### 2.6. Effect of the Mutations on the Protein Post-Fusion Conformation

**Figure 4:**
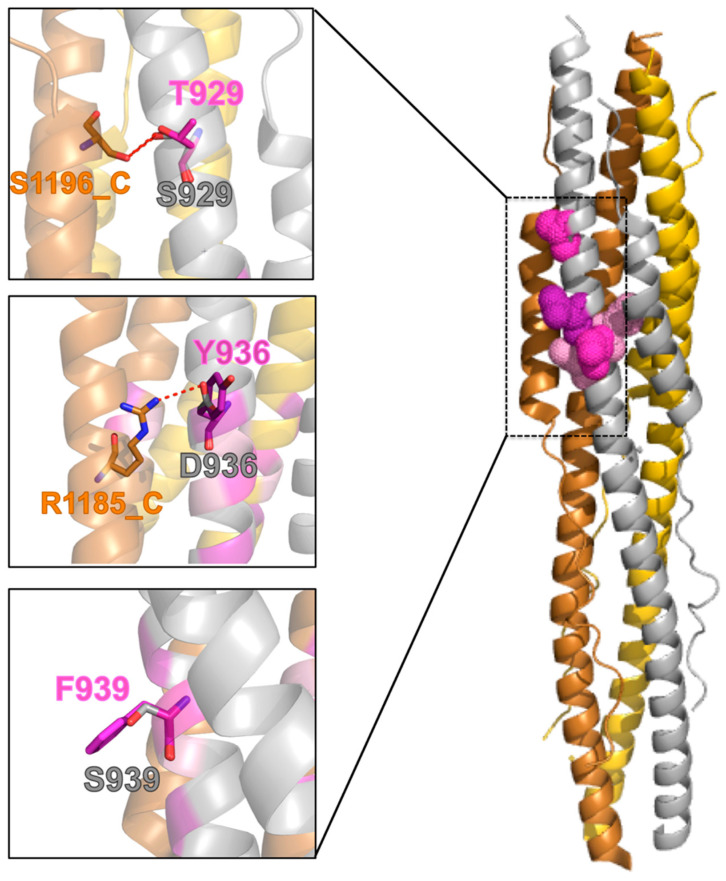
Mutants in the post-fusion conformation. Right: Cartoon representation of the SARS-CoV-2 S protein in its post-fusion trimeric conformation (the three monomers are colored in silver, gold, and copper, PDB ID: 6LXT). The color code is the same in Figure 3. Mutations in the HR1 fusion core are shown in a “dots” representation for chain A. Left: Focus on the structural context of each wild-type residue (silver sticks) and corresponding mutant (purple sticks). H-bonds are shown as red, dashed lines.

When looking at the post-fusion conformation of the SARS-CoV-2 spike protein S2 subunit, these mutations appear more revealing. Two of the wild-type residues, S929 and D936, are engaged in side-chain to side-chain H-bonds with the HR2 segment of an adjacent monomer. In particular, S929 and D936 (HR1 on Chain A) are H-bonded to S1196 and R1185, respectively (HR2 on Chain C, Figure 4). Mutation of S929 to threonine does not cause the loss of the inter-monomer H-bond (Figure 4), while a mutation of D936 to tyrosine, does. The H-bond between D936 and R1185 is actually a salt bridge (estimated to contribute an additional 3–5 kcal/mol to the free energy of protein stability as compared to a neutral H-bond [35]).

Of the remaining most frequent mutations, S939F is completely exposed to the solvent and, therefore, like in the pre-fusion conformation, expected to act unfavorably on the protein solvation energy.

### 2.7. Molecular Dynamics Analysis

When comparing the effect of the mutations on the pre-fusion and post-fusion structures, it emerges that the D936Y mutation is the one expected to have the greatest structural impact. Since it is also the most frequent mutation occurring on the fusion core of S HR1, we decided to further analyze the effect of such a mutation on the structure and dynamics of the SARS-CoV-2 S protein. To this aim, three 0.5-μs long MD simulation replicates were run on the mutant and the wild-type protein, both in their pre-fusion and post-fusion conformations, for a total of 6 μs. We recall in the following the main findings of the MD analysis, while details are reported in the Supplementary Information text and in Figures S1–S12 and Tables S2 and S3.

**Figure 5:**
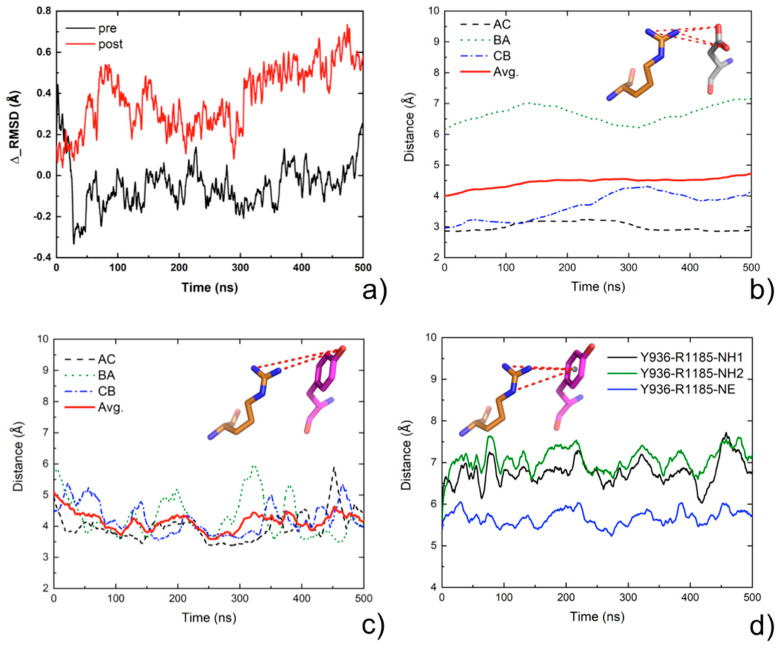
Comparative MD analysis of the Wuhan reference S protein and the D936Y mutant. (**a**) rmsd difference (rmsd) between the wild-type and the mutant in the pre-fusion (black) and post-fusion (red) conformation, averaged over the three independent 500-ns simulations per system. (**b**) Wild-type: minimum distance over time between the carboxylate oxygens of D936 and the guanidinium nitrogens of R1185 on the adjacent monomer, averaged over the three independent simulations per system. Values per single trimer interfaces are plotted as dashed lines while the average values over the three interfaces are plotted as a continuous red line. (**c**) Mutant: minimum distance over time between the hydroxyl oxygen of Y936 and the guanidinium nitrogen of R1185 on the adjacent monomer, averaged over the three independent simulations per system. Values per single trimer interfaces are plotted as dashed lines while the average values over the three interfaces are plotted as a continuous red line. (**d**) Mutant: distances over time between the center of mass of the Y936 aromatic ring and the guanidinium nitrogen of R1185 on the adjacent monomer, averaged over the three independent simulations and the three interfaces.

Both the wild-type and mutant conformations were stable during the whole dynamics, in the pre-fusion and post-fusion conformations, with maximal root mean square deviation (rmsd) values on the Cα atoms not exceeding 3.5 Å from the initial structure (Figures S1 and S7). The difference in the rmsd values between the wild-type protein and the D936Y mutant (Figure 5a) is negligible for the pre-fusion conformation, 0.05 (±0.1) Å. In the post-fusion conformation, the average rmsd is instead higher, by 0.38 Å (±0.2), for the mutant, which seems to acquire some flexibility. The total number of inter-monomer H-bonds from the wild-type to the mutant decreased more in the post-fusion conformation, −1.8 (±1.1), than in the pre-fusion one, −0.9 (±1.3). As we expected, in order for these lost H-bonds to be the inter-monomer D936-R1185 salt bridges we discussed before, we monitored the H-bond distances between D/Y936 and R1185 over time (Figure 5b,c). The minimum distance between the nitrogen atoms of the arginine guanidinium group and the oxygens of the aspartate carboxylate or the hydroxyl oxygen of the mutated tyrosine is reported for each trimer interface. In case of the wild-type, the minimum H-bond distance is 3.32 (±0.7) Å and 3.62 (±1.0) Å for two interfaces, with distances being within 3.5 Å in 70% and 57% of frames, respectively. Therefore, these two H-bonds are largely maintained over time. For the third interface, the average distance is instead 6.48 (1.1) Å, with only 1% of the frames within 3.5 Å. This is consistent with the reference X-ray structure, where D936 and R1185 on the adjacent monomer are at an H-bond distance for two interfaces, and are, instead, 4.71 Å apart on the third interface. In case of the mutants, the average distances are all around 4 Å (3.96 ± 0.7, 4.29 ± 0.9 and 4.23 ± 0.9 Å for each interface), with the total frames featuring a distance within 3.5 Å amounting to only 22%. This correlates with the loss of ≈2 H-bonds in the mutant conformation. However, it is worth it to remind here that, due to its strong electrostatic nature, a stabilizing interaction between D936 and R1185 is maintained above the classical threshold for an H-bond distance [36].

Since an arginine can involve a tyrosine in a cation-π interaction, we also monitored the minimum distance between the nitrogen atoms of the R1185 guanidinium group and the center of mass of the Y936 aromatic ring (Figure 5d). Average values are in the 6–7 Å range and never drop below 4.3 Å, which is considered a reasonable cutoff distance for establishing a cation-π interaction [37]. Therefore, the above analysis ruled out the possibility of having a cation-π interaction between these two residues.

Finally, we followed the buried surface area over the simulation time within the MDcons approach finding the post-fusion assembly to be, overall, more compact (i.e., featuring a moderately higher buried surface area upon complex formation) for the wild-type system, as compared to the D936Y mutant (see Figure S13).

## 3. Discussion

We monitored the mutations accumulated over time on the SARS-CoV-2 S protein HR1 fusion core, and a key structural and functional motif for the virus infectivity, using GISAID as the resource of genomic sequences. The SARS-CoV-2 HR1 fusion core differs in several positions from that of SARS-CoV and its peculiarity has been associated with the higher infectivity of the virus [29]. On 15 February 2021, D936Y was the most frequent mutation on the HR1 fusion core, followed by S939F and S929T. Notably, most of the HR1 fusion core positions are virtually unaffected by mutational events, while all three most-frequent mutations are located on the second of four non-coaxial helical segments composing the HR1. In the pre-fusion conformation, two of these mutations result in large aromatic residues of a tyrosine and a phenylalanine. Such mutations, mainly localized in Europe and USA, are quite late ones, emerging starting from the end of February 2020, and are associated with the late G/GH/GR/GV clades, implying that they co-exist with the globally dominant D614G mutation.

D936Y was the most frequent among the HR1 fusion core mutations on 25 February 2021. While the geographical distribution of S929T, mostly from England, and of S939F, mostly from England, USA, and Denmark, may reflect the higher contribution of these countries to the genomic sequencing of SARS-CoV-2 (the three countries together covered roughly two-thirds of the sequences in GISAID on 15 February), D936Y was widespread. Besides the above countries, in Scandinavia and especially in Finland and Sweden, it represents 17% and 12%, respectively, of all the sequences available from these countries.

We investigated the structural basis of such mutations, finding out that the D936Y mutation is the one expected to have the greatest structural impact. Therefore, we analyzed the effect of such a mutation by molecular dynamics, showing that it causes the loss of a strong inter-monomer salt bridge in the post-fusion conformation of the S protein and introduces some flexibility in it, resulting in an overall slightly reduced compactness of the assembly.

Experimental testing of the D936Y mutation, within a study comprising over 100 S protein variants or glycosylation site modifications [38], has shown a significant decrease of infectivity as compared to the Wuhan reference strain [1] when it was the only variant. It demonstrated instead increased infectivity, as compared to the reference strain, when associated with the D614G variant, which was comparable to that of the strain presenting only the D614G mutation. It is worth noticing that, for other frequent variants included in the same study, such as L5F and D839Y, infectivity was virtually unchanged. The structural effect of the D936Y mutation, that we report here, may call for further functional and clinical studies to clarify its possible consequences on the SARS-CoV-2 virulence.

An up-to-date count of the above mutations is provided at: https://www.molnac.unisa.it/BioTools/cov2smt/index.php (accessed on 18 April 2021).

## 4. Methods

### 4.1. Identification of Mutations

We downloaded the 550,092 genomic sequences available from GISAID on 15 February 2021. From these sequences, we extracted the nucleotide sequences of the spike protein and translated them to protein sequences with in-house scripts. Nucleotide sequences featuring an internal stop codon or having at least one undefined (“N”) nucleotide were discarded. Sequences annotated as pangolin, bat, or canine were also discarded. The remaining 415,673 protein sequences were further analysed. As a reference system, we used the genomic sequence with GISAID ID: EPI_ISL_402124, isolated and sequenced in Wuhan (Hubei, China) on 30 December 2019 [1]. Then, upon alignment to the reference sequence, we identified point mutations in all the sets of at least two sequences.

The web application was built using standard HTML, php, and python scripts.

### 4.2. Mutants Modelling and Analysis

Mutants 3D models were built using the mutate_model module of the Modeller 9v11 program [39]. This is an automated method for modelling point mutations in protein structures, which includes an optimisation procedure of the mutated residue in its environment, beginning with a conjugate gradients’ minimisation, continuing with molecular dynamics with simulated annealing, and finishing again by conjugate gradients. The used force field is CHARM-22. For details, see Reference [40]. Models for mutants in the pre-fusion conformation were built starting from the EM structure of the pre-fusion trimeric conformation (PDB ID: 6VSB, resolution 3.46 Å, [22]). Models for mutants in the post-fusion conformation were built starting from the X-ray structure of the S2 subunit fusion core, featuring residues 912-988 and 1164-1202 (PDB ID: 6LXT, resolution 2.90 Å, [29]). Molecular models were analysed and visually inspected with Pymol [41]. The COCOMAPS web server [42] was used to analyse the inter-chain contacts and H-bonds as well as the residues accessibility to the solvent.

### 4.3. Molecular Dynamics Simulations

Molecular dynamics simulations were carried out for the wild-type S protein and for the D936Y mutant in the pre-fusion and post-fusion conformations, starting from the experimental structures used for modeling the mutants (see above). For the pre-fusion simulations, we used the trimer of the S2 subunit (PDB ID: 6VSB). From S711 to C1146, respectively, 200 residues upstream and ≈160 residues downstream of HR1. Missing residues between K811 and R815 and between L828 and Q853 were modeled with the GalaxyFill program [43]. The crystal structure of the post fusion core of the protein S2 subunit (PDB ID: 6LXT), featuring residues 912-988, 1164-1202 [29] was used for the post-fusion simulations. For the D936 mutant, models obtained as detailed in the previous section were used.

All the MD simulations were carried out with Gromacs 2018 [44], using the Amber14SB force field [45]. Each protein was inserted into a rectangular box of TIP3P water molecules, setting a minimum distance of 12.0 Å from it to the box sides and neutralizing the solution with Zn^2+^ and Cl^−^ ions. A minimization was first carried out, followed by isothermal ensemble (NVT) dynamics using a velocity-rescale thermostat [46] for computing positions and velocities of atoms. Then, 2 ns of isothermal-isobaric ensemble (NPT) dynamics was carried out to equilibrate the structure. Periodic boundary conditions were applied in all directions. The production simulations were carried out using an NPT ensemble for 500 ns. The temperature was maintained constant at 300 K using a velocity-rescale thermostat [46] (τ_T_ = 0.1 ps) and a pressure of 1 bar was maintained using a Parrinello-Rahman barostat [47] (τ_P_ = 2.0 ps). Electrostatic interactions beyond 1.2 nm were evaluated by the Particle-Mesh-Ewald (PME) method [48]. Bond lengths were constrained with the LINear Constraint Solver algorithm [49]. Trajectories were analyzed using Gromacs 2018 analysis tools.

For the MDcons analyses [50], using a contact-based approach [51,52] for the dynamical characterization of the interface in protein assemblies, 500 snapshots were generated for each system, by writing the coordinates every 1 ns.

## Figures and Tables

**Table 1:** Occurrences of most frequent mutations on the HR1 “fusion core” on 15 February 2021.

# S Protein Sequences	S929T	D936Y	S939F
415,673	467	1296	1108

**Table 2:** Solvent accessibility of mutated residues in the pre-fusion and post-fusion conformations.

Amino Acid	Pre-Fusion	Post-Fusion
T929	exposed	partly buried (18.6%) ^a^
Y936	exposed	partly buried (19.0%)
F939	exposed	exposed

^a^ Percentage of buried surface upon complex formation.

## Data Availability

The data presented in this study are available in supplementary material.

## Supplementary Materials

Table S1. Number of occurrences of all mutations on the HR1 “fusion core” on 15 February 2021. Table S2. MD analysis data of the wt and D936Y mutant pre-fusion state. Table S3. MD analysis data of the wt and D936Y mutant post-fusion state. Figures S1–S2. Pre-fusion state: Cα RMSD values versus time. Figure S3. Pre-fusion state: RMSF values per residue. Figure S4. Pre-fusion state: Average number of hydrogen bonds versus time. Figure S5. Pre-fusion state: Potential energy versus time. Figure S6. Pre-fusion state: Electrostatic (ELE) and Lennard Jones (LJ) energies versus time. Figures S7–S8. Post-fusion state: Cα RMSD values versus time. Figure S9. Post-fusion state. RMSF per residue. Figure S10. Post-fusion state: Average number of hydrogen bonds versus time. Figure S11. Post-fusion state: Potential energy versus time. Figure S12. Post-fusion state: Electrostatic (ELE) and Lennard Jones (LJ) energies versus time. Figure S13. Buried surface area along the MD simulations for the wt and D936Y mutant post-fusion state.
