# Supplementary material for: D936Y and Other Mutations in the Fusion Core of the SARS-CoV-2 Spike Protein Heptad Repeat 1: Frequency, Geographical Distribution, and Structural Effect
Source: Molecules. 2021 Apr 30;26(9):2622. doi: 10.3390/molecules26092622 (PMC8124767; doi:10.3390/molecules26092622)
Supplement: Supplementary file 1 [file molecules-26-02622-s001.zip › molecules-1167936-SI.pdf]

Supplementary Materials for:

"*D936Y and Other Mutations in the Fusion Core of the hCov-19 Spike Protein Heptad Repeat 1 Affect the Post-Fusion Assembly*", by Romina Oliva[1], Abdul Rajjak Shaikh[2], Andrea Petta,[3] Anna Vangone,[4] Luigi Cavallo[2]

[1]Department of Sciences and Technologies, University Parthenope of Naples, Centro Direzionale Isola C4, I-80143, Naples, Italy.
[2]King Abdullah University of Science and Technology (KAUST), Physical Sciences and Engineering Division, Kaust Catalysis Center, Thuwal 23955-6900, Saudi Arabia.
[3]Dipartimento di Informatica ed Applicazioni, University of Salerno, Via Papa Paolo Giovanni II, I-84048, Fisciano, Italy.
[4]Pharma Research and Early Development, Large Molecule Research, Roche Innovation Center Munich, Nonnenwald 2, 82377 Penzberg, Germany

Email: romina.oliva@uniparthenope.it

Table S1. Occurrences of all non synonymous mutations on the HR1 “fusion core” of SARS-CoV-2 S protein, on February 15th 2021. For each position the wild-type amino acid (wt-aa) is reported in the 2nd column and the mutated amino acids (Mut-aa) with relative occurrences (Occ) are reported in the following columns.

Position	Wt-aa	Mut-aa	Occ	Mut-aa	Occ	Mut-aa	Occ	Mut-aa	Occ	Mut-aa	Occ	Mut-aa	Occ	Mut-aa	Occ	Mut-aa	Occ	Mut-aa	Occ	%1st mut	%all mut
929	**S**	**T**	467	I	60	N	5	R	1	G	1									0,1123	0,1285
930	A	V	79	S	4	T	1	K	1											0,0190	0,0204
931	I	V	3	A	3	T	1													0,0007	0,0017
932	G	S	202	C	9	V	8	D	5	I	3	R	1	Q	1					0,0485	0,0551
933	K	G	3	R	1	D	1													0,0007	0,0012
934	I	V	7	M	5	L	5	K	3	S	1									0,0016	0,0050
935	Q	I	3	H	1	R	1	E	1	L	1									0,0007	0,0017
936	**D**	**Y**	1296	N	148	H	125	V	44	G	24	E	17	Q	3	A	1	S	1	0,3117	0,3991
937	S	L	28	T	4	D	3	A	1											0,0067	0,0087
938	L	F	227	I	26	S	3	T	1											0,0546	0,0618
939	**S**	**F**	1108	Y	5	L	3	A	1											0,2665	0,2687
940	S	F	174	A	7	Y	6	T	3											0,041	0,045

														9	7
941	T	A	48	I	11	-	4	S	3					0,0115	0,0159
942	A	S	105	T	82	V	10	E	1	L	1	-[a]	1	0,0253	0,0481
943	S	P	144	I	26	G	4	T	4	R	3	-	1	0,0346	0,0438
944	A	V	1	K	1	-	1							0,0002	0,0007
945	L	I	2	F	2	-	1							0,0005	0,0012
946	G	V	69	E	7	R	4	Q	1					0,0166	0,0195
947	K	R	44	I	1	D	1							0,0106	0,0111
948	L	I	12	R	1	V	1							0,0029	0,0034
949	Q	R	2	L	1	V	1							0,0005	0,0010

[a] "-" represents a gap at that position.

Detailed results of the MD simulations

Pre-fusion conformation

The average RMSD values calculated on the Cα atoms for the wild-type S protein and the D936Y mutant in the pre-fusion conformation are 3.19 (±0.2) and 3.14 (±0.1) Å, respectively, resulting marginally higher (by 0.05 Å) for the wild-type system (Figures S1-S2). Root mean square fluctuation (RMSF) profiles, shown in Figure S3, are also comparable for the two systems and exhibit, not surprisingly, the highest values on the segment 808-813 and 829-856 corresponding to flexible loops whose coordinates, modeled by us for the sake of the simulations stability, are mostly missing from the experimental structure. The inter-monomer hydrogen bonds analysis also does not show significant differences between the two systems, as shown in Figure S4 and Table S2. As for potential energy, the obtained average values are comparable, -4,252.67 ± 1.3 x 10^3 kJ/mol for the wild-type and -4,249.95 ± 1.3 x 10^3 kJ/mol for the mutant (Figures S2). Finally, the interaction energy between monomers is higher in the mutant, with the Lennard Jones contribution in particular being ≈50 kJ/mol higher (Figure S6 and Table S2). This was the most significant difference between the wild-type and D936Y mutant in the pre-fusion conformation highlighted by the MD simulations.

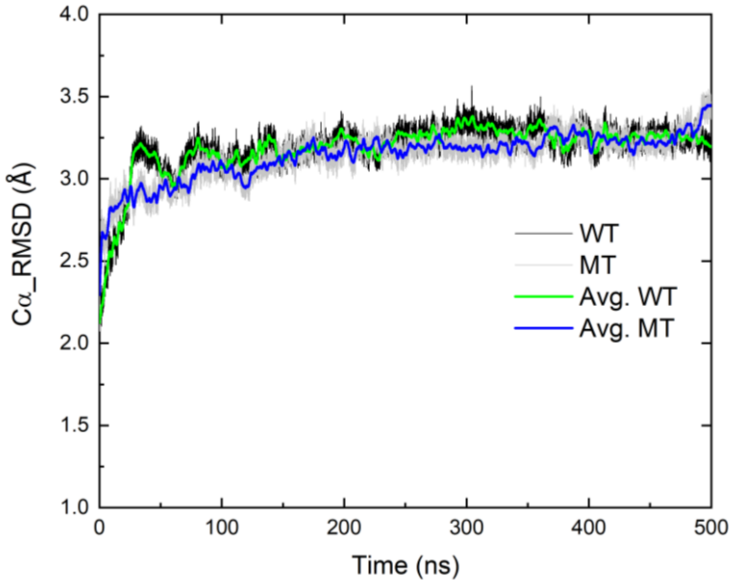

Figure S1. Cα RMSD values shown as a function of time for the wild-type S2 protein (WT, black/green) and the D936Y mutant (MT, gray/blue) in the pre-fusion conformation, averaged over the three simulations and three monomers.

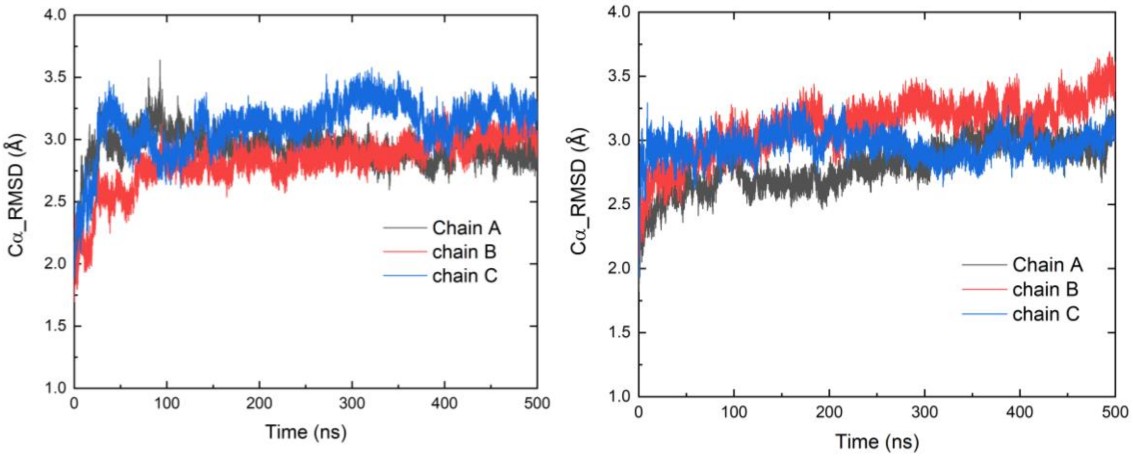

(A) (B)

Figure S2. Cα RMSD values as a function of time for the wild-type S protein (A) and the D936Y mutant (B) reported for each monomer in the pre-fusion conformation, averaged over the three simulations.

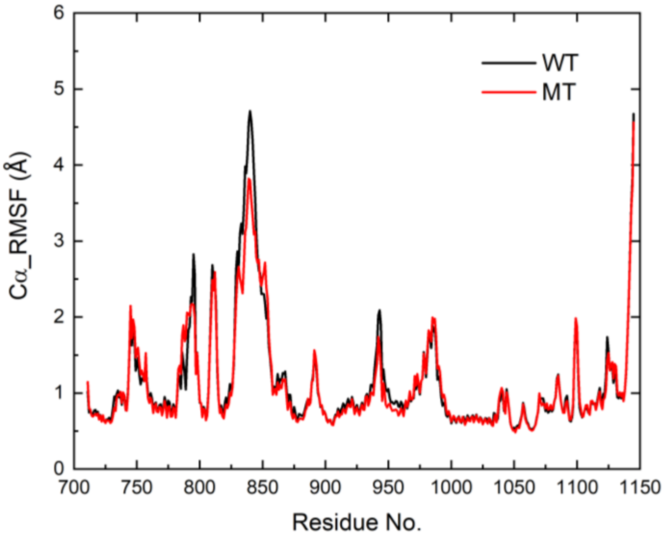

Figure S3. Root mean square fluctuation values versus the residue numbers for the wild-type S protein and D936Y mutant in the pre-fusion conformation.

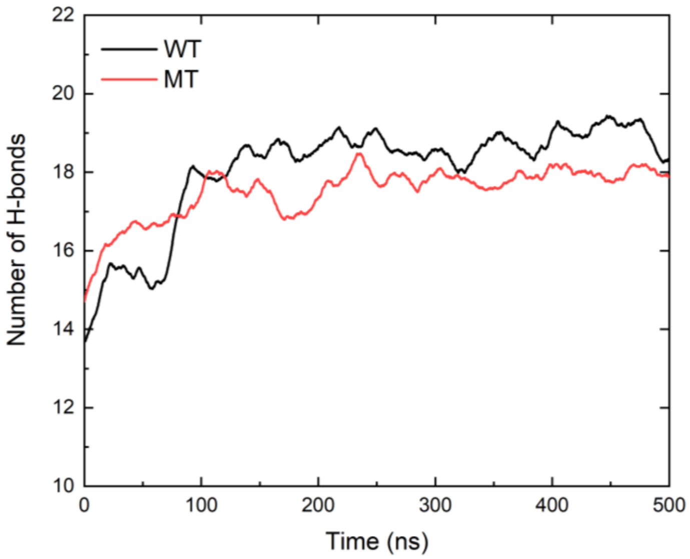

Figure S4. Average number of hydrogen bonds against time in the pre-fusion conformation of wild-type and mutant compounds.

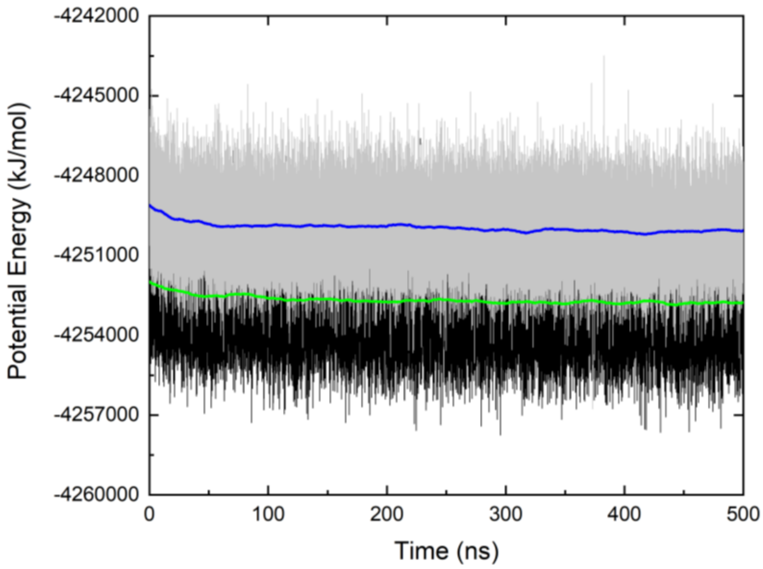

Figure S5. Potential energy versus time, averaged over the three simulations. Values for the wild-type S protein and the D936Y mutant are shown as black and gray lines, respectively. Average values are shown as green and blue lines for the wild-type and mutant, respectively.

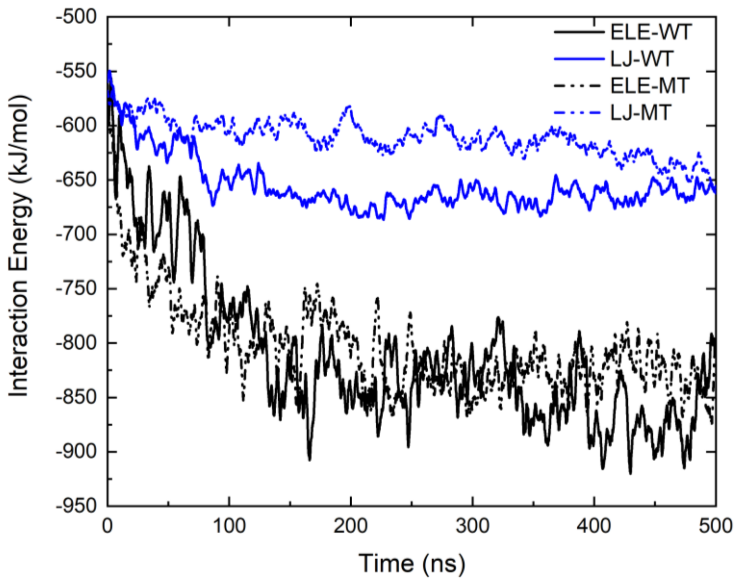

Figure S6. Electrostatic (ELE) and Lennard Jones (LJ) components of the interaction energy monitored over time in the pre-fusion conformation of the for wild-type S protein and the D936Y mutant.

Table S2. Pre-fusion state. Average number of hydrogen bonds between different monomers, and average interaction energy between different monomer due to Coulomb interactions, E_{El}, and Lennard Jones, E_{LJ}, interactions. The standard deviations are shown in parentheses.

System	#H-bonds	E_{El}	E_{LJ}
WT	18.8 (±1.0)	-848 (±40)	-667 (±13)
MT	17.9 (±0.8)	-828 (±33)	-619 (±17)
delta	-0.9	+20	+48

Post-fusion conformation

The average RMSD values calculated on the Cα atoms for the wild-type S protein and the D936Y mutant in the post-fusion state are 2.08 (±0.1) and 2.46 (±0.2) Å, respectively (Figures S7-S8). Root mean square fluctuation (RMSF) profiles, shown in Figure S9, are similar for the two systems, with that of the mutant being slightly higher.

The average number of total inter-monomer H-bonds over time for each system are reported in Table S3 and plotted in Figure S10. It is lower by 2 units in the mutant, because of the loss of H-bonds between D936 (mutated to Y936) and R1185 on an adjacent monomer, as discussed in the main text.

The potential energy over simulation time is plotted in Figure S11. It remains stable throughout the simulations. Regarding the interaction energy, in the mutant it is 101 kJ/mol higher than in the wild-type system. This energy difference is due to the electrostatic term, while the Lennard Jones term doesn't change significantly, as shown in Figure S12. This appears clearly to be the consequence of the loss of inter-monomer salt bridge interactions, as discussed in the main text.

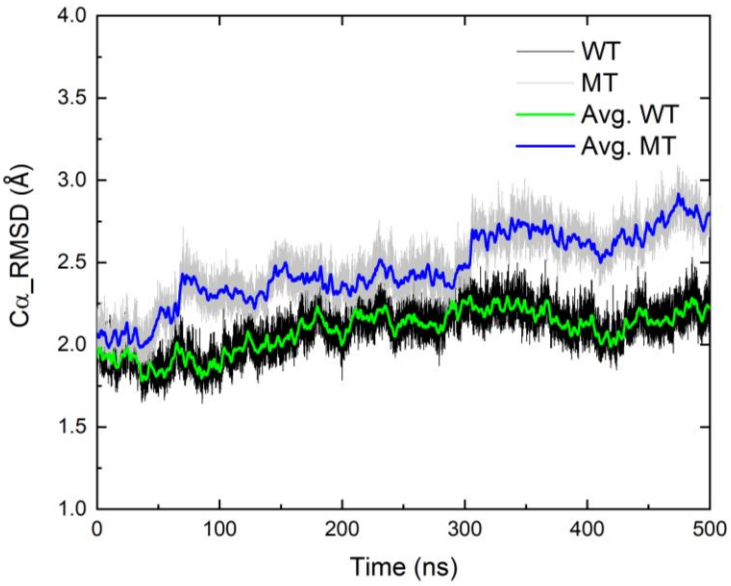

Figure S7. Cα RMSD values shown as a function of time for the wild-type S2 protein (WT, black/green) and the D936Y mutant (MT, gray/blue) in the post-fusion conformation, averaged over the three simulations and three monomers.

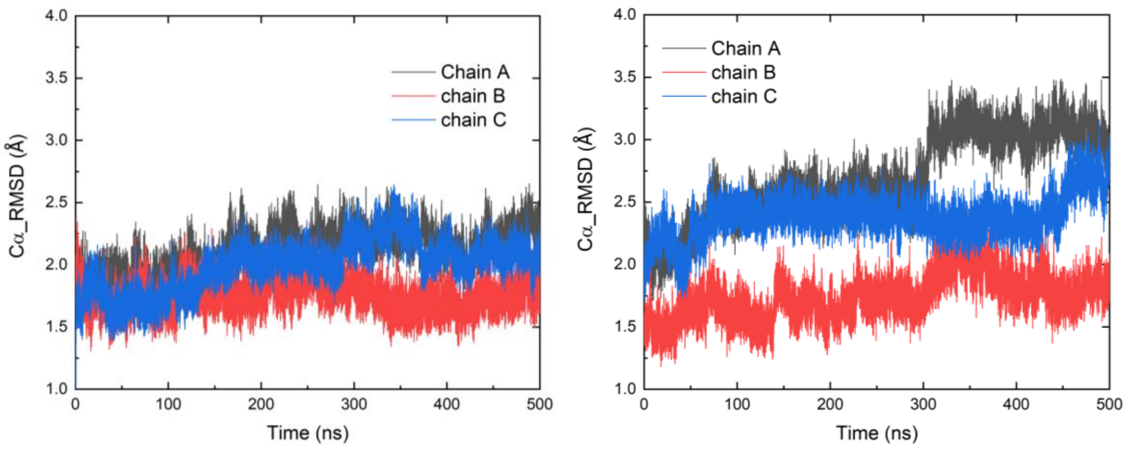

(A) (B)

Figure S8. Cα RMSD values as a function of time for the wild-type S protein (A) and the D936Y mutant (B) reported for each monomer in the post-fusion conformation, averaged over the three simulations.

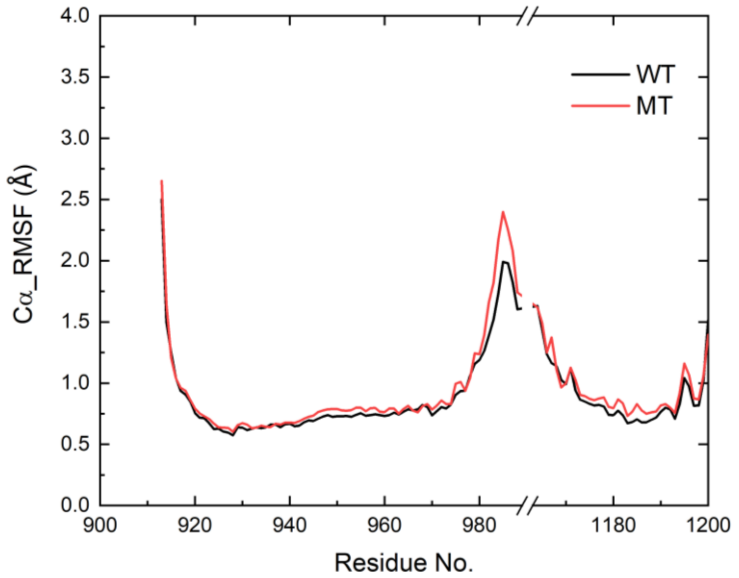

Figure S9. Root means quare fluctuation versus residue numbers for the wild -type (black color) and mutant (gray color) protein in the post-fusion conformation.

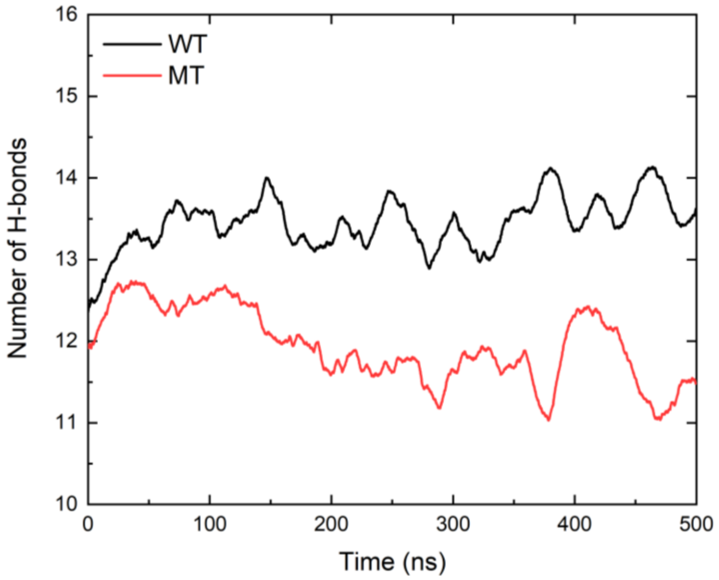

Figure S10. Average number of hydrogen bonds versus simulation time for the post-fusion conformation of wild-type S protein and the D936Y and mutant.

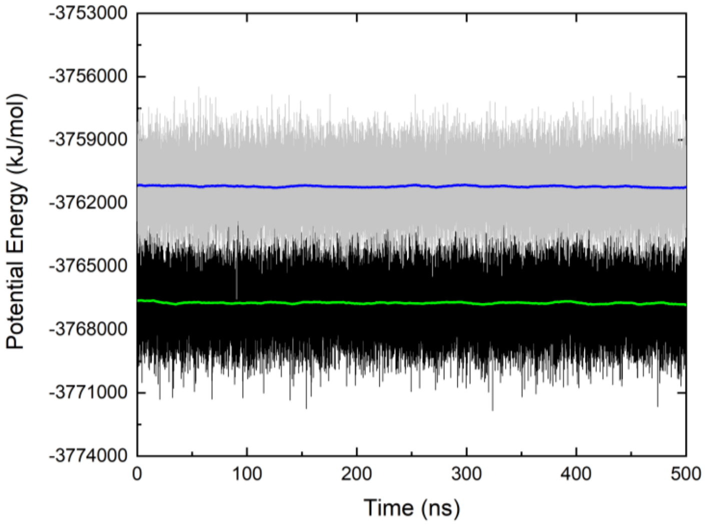

Figure S11. Potential energy values versus time are shown as black and gray line for the wild-type and mutant, respectively, with average values shown as green and blue lines.

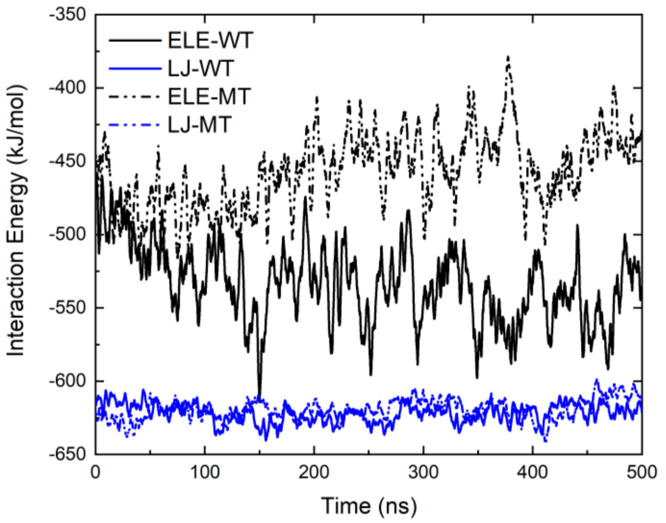

Figure S12. Interaction energy due to electrostatic (ELE) and Lennard Jones (LJ) interaction with time in wild-type (solid lines) and mutant (dotted lines).

Table S3. Post-fusion state. Average number of hydrogen bonds between different monomers, and average interaction energy between different monomer due to Coulomb interactions, E_{El}, and Lennard Jones, E_{LJ}, interactions. The standard deviations are shown in parentheses.

**System**	**#H-bonds**	**E_{El}**	**E_{LJ}**
WT	13.5 (±0.8)	-542 (±33)	-622 (±11)
MT	11.7 (±0.7)	-445 (±31)	-618 (±12)
delta	-1.8	+97	+4

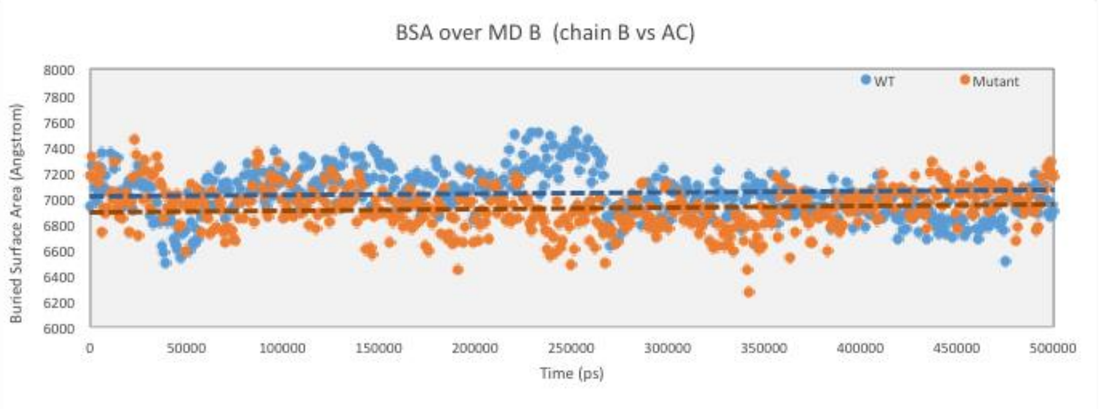

Figure S13. Buried surface area over simulation time of chain B versus chains A and B for the post-fusion state of the wild-type (light blue) and mutant systems (orange), calculated by NACCESS within the MDcons package. Average values (7024 and 6916 Å^2, respectively) are shown with dotted lines.
